# Supplementary material for: Human immunoglobulin gene allelic variation impacts germline-targeting vaccine priming
Source: NPJ Vaccines. 2024 Mar 11;9:58. doi: 10.1038/s41541-024-00811-5 (PMC11384754; doi:10.1038/s41541-024-00811-5)
Supplement: Supplementary file 1 — Supplementary Material [file 41541_2024_811_MOESM1_ESM.pdf]

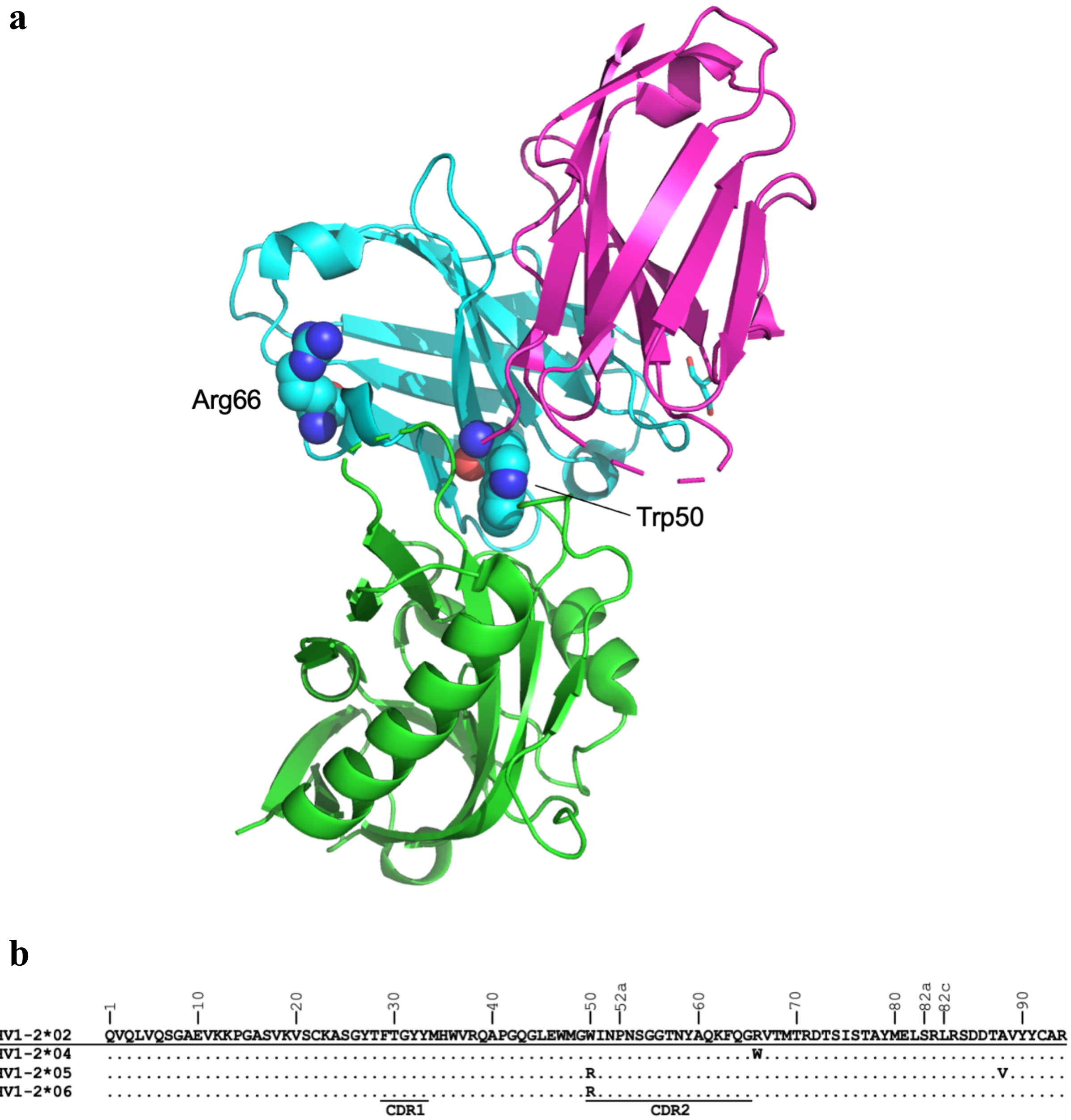

Supplementary Figure 1. Locations of key allele-defining mutations in the structure and sequence of IGHV1-2. (a) Structural model of eOD-GT8 (green) bound to a VRC01-class human naive precursor (cyan, purple), based on PDB: 5IES. Antibody heavy chain residues Trp50, present in IGHV1-2*02 but mutated to Arg50 in IGHV1-2*05 and *06, is shown in spheres and labelled. Trp50, which is highly conserved across VRC01-class bnAbs (the only exception being the bnAb 12A21 with Leu50) [9,10,12,23,52-54], interacts with the highly conserved HIV gp120 residue Asn280 [10-12,53-55] (not shown) and is generally considered a canonical part of the VRC01-class interaction with HIV Env. Antibody heavy chain residue Arg66, present in IGHV1-2*02 but mutated to Trp66 in IGHV1-2*04, is shown in spheres and labelled. **(b)** IGHV1-2 sequence alignment showing alleles confirmed by single nucleotide polymorphism (SNP) analysis and other methods [15].

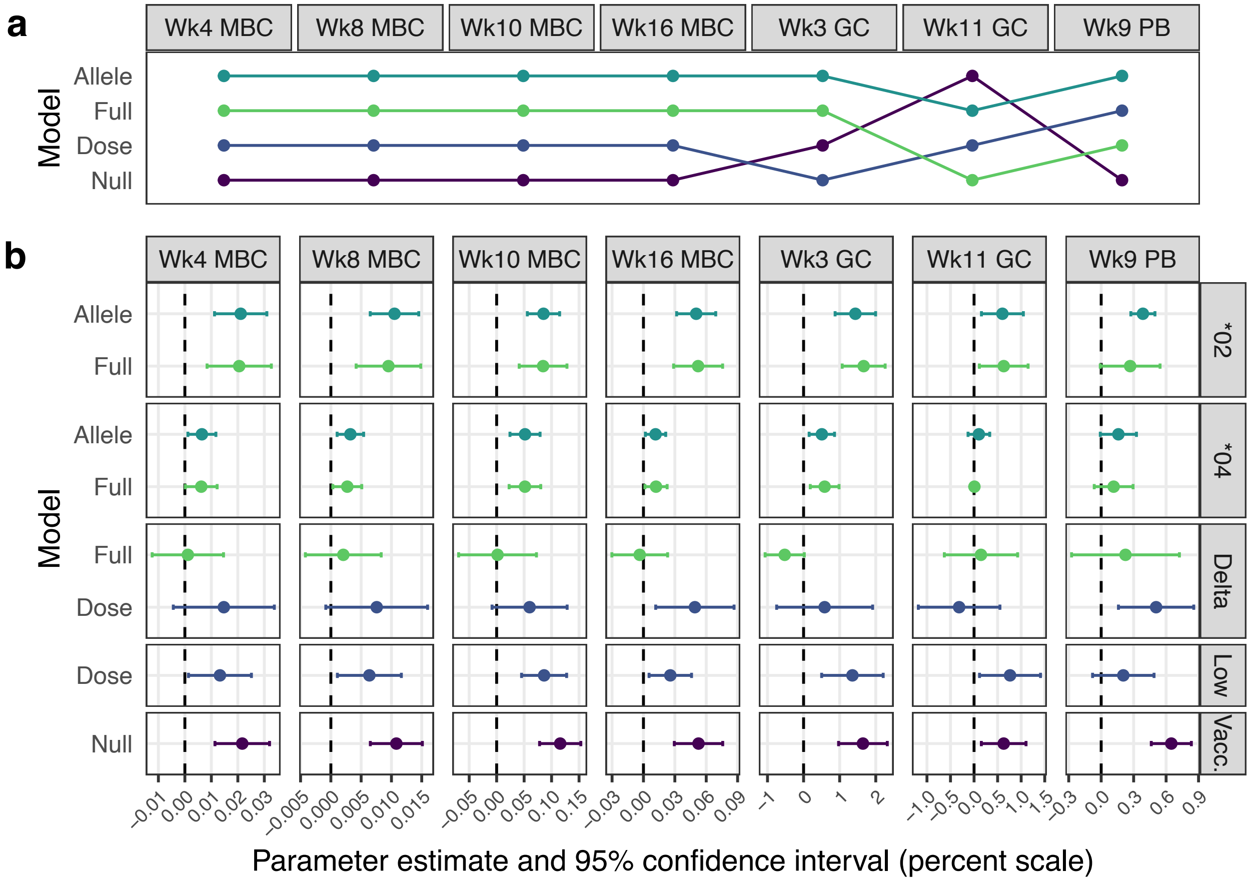

Supplementary Figure 2. Statistical modeling of post-vaccination VRC01-class B cell frequencies. (a) Model rankings from QAICc (see text and Methods) are shown as best to worst, from top to bottom, for each sample type and timepoint shown left to right. For all MBC timepoints, models were ranked in the same order from best to worst: (i) Allele, (ii) Full, (iii) Dose, and (iv) Null. Each color-coded line connects the ranking of the models across time points. **(b)** Points and lines show estimates and 95% confidence intervals (CIs) for each model parameter, for different models labelled on the y axis, at each time point labeled at the top of the graph. Model parameters representing the per-allele contributions of *02 and *04, the contributions of dose delta (100µg - 20µg) and dose=20µg, and a single estimate for the vaccine dose groups pooled (Null model) are grouped in rows *02, *04, Delta, Low and Vacc., respectively, as indicated on the right side of the panel. Parameter estimates and CIs are color-coded by model as in panel (a) and are displayed with values on the percent scale as indicated by the x-axis.

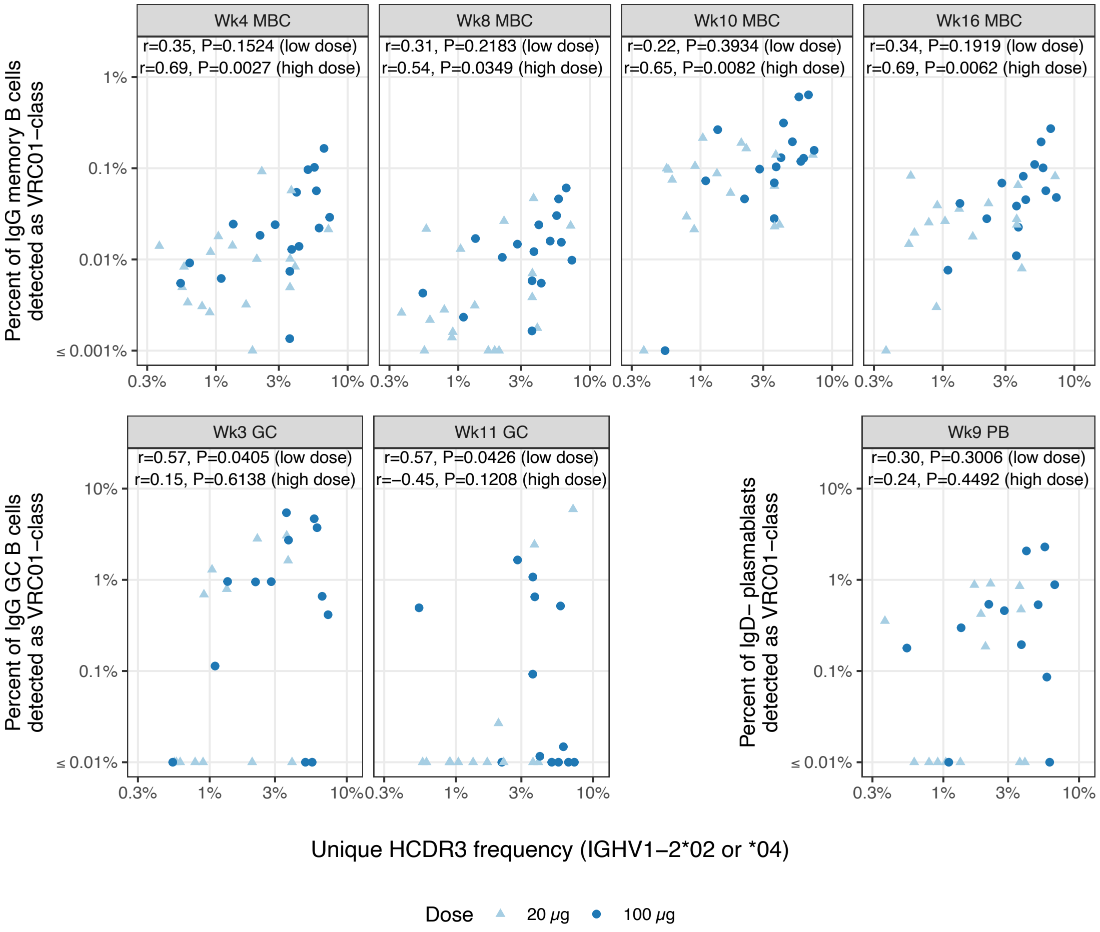

Supplementary Figure 3. Correlations between pre-vaccination IgM unique HCDR3 frequency (IGHV1-2*02 or *04) and the percent VRC01-class B cell response by visit and treatment group. Points are shape- and color-coded as shown in the legend. Spearman correlation coefficient (r) and P-values are displayed for each time point and dose group.

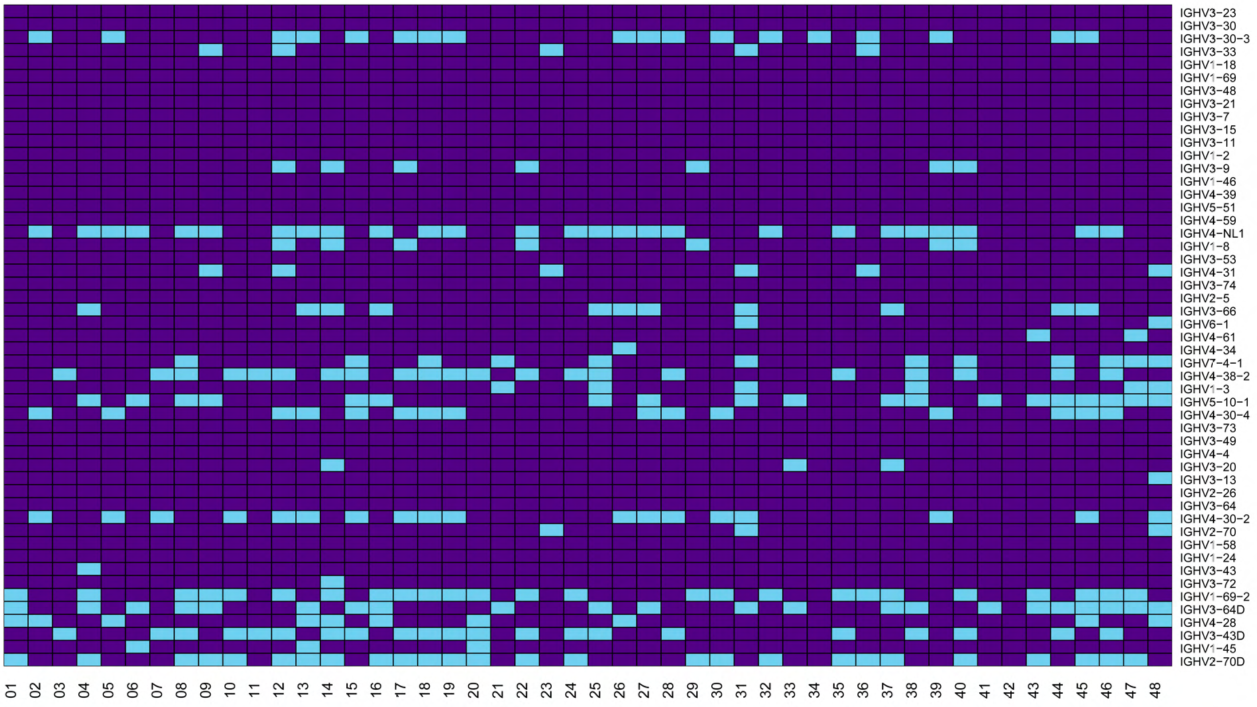

Supplementary Figure 4. Captured IGHV genes in each of the 48 IAVI G001 trial participants. The x-axis shows the G001 trial participant cases, and the y-axis shows the IGHV genes in the order they are found in typical IgM repertoires, with the most utilized genes at the top and the least utilized genes at the bottom. Captured (dark blue) and not captured (light blue) genes are indicated for each case.

Supplementary Table 1: IGHV1-2 genotype counts by treatment group.

Treatment	*02/*02	*02/*04	*02/*05	*02/*06	*04/*04	*04/*05	*04/*06	*05/*06
100 µg	7	4	2	0	1	1	2	1
20 µg	1	4	0	0	7	1	5	0
Placebo	3	5	0	2	2	0	0	0
Total	11	13	2	2	10	2	7	1

Supplementary Table 2: Frequency of IGHV1-2 mRNA expression in the naive repertoire. The number of trial participants (n), mean, 95% confidence interval (95% CI), minimum (Min) and maximum (Max) are reported for groups defined by homozygous or heterozygous genotypes including each allele. For participants homozygous for an allele, the per-allele frequency is defined as half the measured frequency of that allele. For *02/*02_S4953 participants, the per-allele usage frequency is defined as half of the sum of the *02 and *02_S4953 frequencies and the participant is classified as a *02 homozygote.

	Allele	n	Mean	95% CI	Min	Max
*02	Homozygote	11	3.15%	2.72% to 3.57%	2.28%	4.52%
	Heterozygote	17	3.34%	2.87% to 3.82%	1.98%	6.29%
*04	Homozygote	10	0.86%	0.67% to 1.06%	0.41%	1.22%
	Heterozygote	22	0.72%	0.63% to 0.81%	0.25%	1.12%
*05	Heterozygote	5	0.09%	0.03% to 0.14%	0.03%	0.14%
*06	Heterozygote	10	2.43%	1.87% to 2.99%	1.78%	4.18%

SupplementaryTable 3: Estimated difference in the mean per-allele frequency of IGHV1-2 mRNA expression between participants homozygotes and heterozygotes for each allele. For participants homozygous for an allele, the per-allele frequency is defined as half the measured frequency of that allele. The 95% confidence interval and P-value are based on a T-test for the null hypothesis that the true difference (mean among homozygous minus mean among heterzygous) is 0%.

Allele	Difference	95% CI	P-value
*02	-0.19%	-0.80% to 0.41%	0.51
*04	0.14%	-0.07% to 0.35%	0.17

Supplementary Table 4: Percent of IgG B cells detected as VRC01-class testing between genotype groups among detectable response participants. Comparisons with at least 2 samples per group are included and P-values displayed for comparisons with at least 3 samples per group. Testing was done using the Wilcoxon rank-sum test. Median, minimum, and maximum values are presented by treatment group and time point. Unadjusted and FDR adjusted P-values displayed.

		Comparison	Sample Sizes	Median (Range)	P Value	
					Unadjusted	FDR Adjusted
**20 µg**						
	Wk4 MBC	*02/*04 vs. *04/*04	4 vs. 6	0.0092 [0.0049, 0.0572] vs. 0.0121 [0.0026, 0.0924]	1.0000	1.0000
		*02/*04 vs. *04/*05 or *04/*06	4 vs. 6	0.0092 [0.0049, 0.0572] vs. 0.0066 [0.0031, 0.0140]	0.4762	0.8081
		*04/*04 vs. *04/*05 or *04/*06	6 vs. 6	0.0121 [0.0026, 0.0924] vs. 0.0066 [0.0031, 0.0140]	0.4848	0.8081
	Wk8 MBC	*02/*04 vs. *04/*04	4 vs. 4	0.0054 [0.0018, 0.0469] vs. 0.0081 [0.0014, 0.0264]	0.8857	0.9797
		*02/*04 vs. *04/*05 or *04/*06	4 vs. 6	0.0054 [0.0018, 0.0469] vs. 0.0024 [0.0007, 0.0216]	0.2571	0.8000
		*04/*04 vs. *04/*05 or *04/*06	4 vs. 6	0.0081 [0.0014, 0.0264] vs. 0.0024 [0.0007, 0.0216]	0.3524	0.8000
	Wk10 MBC	*02/*04 vs. *04/*04	4 vs. 6	0.0440 [0.0230, 0.1404] vs. 0.1263 [0.0214, 0.2148]	0.3524	0.8000
		*02/*04 vs. *04/*05 or *04/*06	4 vs. 6	0.0440 [0.0230, 0.1404] vs. 0.0854 [0.0005, 0.1057]	0.7619	0.9797
		*04/*04 vs. *04/*05 or *04/*06	6 vs. 6	0.1263 [0.0214, 0.2148] vs. 0.0854 [0.0005, 0.1057]	0.3939	0.8000
	Wk16 MBC	*02/*04 vs. *04/*04	4 vs. 5	0.0259 [0.0080, 0.0653] vs. 0.0263 [0.0030, 0.0412]	0.9048	0.9797
		*02/*04 vs. *04/*05 or *04/*06	4 vs. 6	0.0259 [0.0080, 0.0653] vs. 0.0225 [0.0001, 0.0826]	0.9143	0.9797
		*04/*04 vs. *04/*05 or *04/*06	5 vs. 6	0.0263 [0.0030, 0.0412] vs. 0.0225 [0.0001, 0.0826]	0.9307	0.9797
	Wk3 GC	*02/*04 vs. *04/*04	2 vs. 3	2.3429 [1.6281, 3.0577] vs. 1.2946 [0.7917, 2.8275]	—	—
	Wk9 PB	*02/*04 vs. *04/*04	2 vs. 4	0.6629 [0.4731, 0.8527] vs. 0.6510 [0.1856, 0.9081]	—	—
**100 µg**						
	Wk4 MBC	*02/*02 vs. *02/*04	7 vs. 4	0.0566 [0.0139, 0.1649] vs. 0.0185 [0.0014, 0.0545]	0.1091	0.7273
		*02/*02 vs. *02/*05 or *02/*06	7 vs. 2	0.0566 [0.0139, 0.1649] vs. 0.0129 [0.0074, 0.0184]	—	—
		*02/*02 vs. *04/*05 or *04/*06	7 vs. 3	0.0566 [0.0139, 0.1649] vs. 0.0062 [0.0055, 0.0092]	0.0167	0.2424
		*02/*04 vs. *02/*05 or *02/*06	4 vs. 2	0.0185 [0.0014, 0.0545] vs. 0.0129 [0.0074, 0.0184]	—	—
		*02/*04 vs. *04/*05 or *04/*06	4 vs. 3	0.0185 [0.0014, 0.0545] vs. 0.0062 [0.0055, 0.0092]	0.4000	0.8000
		*02/*05 or *02/*06 vs. *04/*05 or *04/*06	2 vs. 3	0.0129 [0.0074, 0.0184] vs. 0.0062 [0.0055, 0.0092]	—	—
	Wk8 MBC	*02/*02 vs. *02/*04	7 vs. 4	0.0159 [0.0055, 0.0608] vs. 0.0134 [0.0016, 0.0240]	0.3152	0.8000
		*02/*02 vs. *02/*05 or *02/*06	7 vs. 2	0.0159 [0.0055, 0.0608] vs. 0.0082 [0.0058, 0.0105]	—	—
		*02/*02 vs. *04/*05 or *04/*06	7 vs. 2	0.0159 [0.0055, 0.0608] vs. 0.0033 [0.0023, 0.0043]	—	—
		*02/*04 vs. *02/*05 or *02/*06	4 vs. 2	0.0134 [0.0016, 0.0240] vs. 0.0082 [0.0058, 0.0105]	—	—
		*02/*04 vs. *04/*05 or *04/*06	4 vs. 2	0.0134 [0.0016, 0.0240] vs. 0.0033 [0.0023, 0.0043]	—	—
		*02/*05 or *02/*06 vs. *04/*05 or *04/*06	2 vs. 2	0.0082 [0.0058, 0.0105] vs. 0.0033 [0.0023, 0.0043]	—	—
	Wk10 MBC	*02/*02 vs. *02/*04	7 vs. 4	0.1951 [0.1188, 0.6376] vs. 0.1007 [0.0281, 0.1305]	0.0242	0.2424
		*02/*02 vs. *02/*05 or *02/*06	7 vs. 2	0.1951 [0.1188, 0.6376] vs. 0.0577 [0.0461, 0.0692]	—	—
		*02/*04 vs. *02/*05 or *02/*06	4 vs. 2	0.1007 [0.0281, 0.1305] vs. 0.0577 [0.0461, 0.0692]	—	—
	Wk16 MBC	*02/*02 vs. *02/*04	7 vs. 4	0.1010 [0.0452, 0.2718] vs. 0.0457 [0.0110, 0.0817]	0.1636	0.8000
		*02/*02 vs. *02/*05 or *02/*06	7 vs. 2	0.1010 [0.0452, 0.2718] vs. 0.0332 [0.0280, 0.0385]	—	—
		*02/*04 vs. *02/*05 or *02/*06	4 vs. 2	0.0457 [0.0110, 0.0817] vs. 0.0332 [0.0280, 0.0385]	—	—
	Wk3 GC	*02/*02 vs. *02/*04	4 vs. 3	2.1944 [0.4144, 4.6746] vs. 2.7265 [0.9546, 5.4440]	0.6286	0.9670
	Wk11 GC	*02/*02 vs. *02/*04	2 vs. 4	0.2658 [0.0148, 0.5167] vs. 0.3717 [0.0116, 1.6519]	—	—
	Wk9 PB	*02/*02 vs. *02/*04	4 vs. 3	0.7086 [0.0858, 2.2943] vs. 0.4593 [0.1946, 2.0745]	0.8571	0.9797

Supplementary Table 5: Percent of IgG B cells detected as VRC01-class testing between treatment groups among detectable response participants. Comparisons with at least 2 samples per group are included and P-values displayed for comparisons with at least 3 samples per group. Testing was done using the Wilcoxon rank-sum test (two-sided, $\alpha = 0.05$) and P-values less than 0.05 are highlighted. Median, minimum, and maximum values are presented by treatment group and time point. Unadjusted and FDR adjusted P-values displayed.

	Comparison	Sample Sizes	Median (Range)	P Value	
				Unadjusted	FDR Adjusted
**02/*04**					
Wk4 MBC	20 µg vs. 100 µg	4 vs. 4	0.0092 [0.0049, 0.0572] vs. 0.0185 [0.0014, 0.0545]	0.8857	0.9048
Wk8 MBC	20 µg vs. 100 µg	4 vs. 4	0.0054 [0.0018, 0.0469] vs. 0.0134 [0.0016, 0.0240]	0.8857	0.9048
Wk10 MBC	20 µg vs. 100 µg	4 vs. 4	0.0440 [0.0230, 0.1404] vs. 0.1007 [0.0281, 0.1305]	0.4857	0.9048
Wk16 MBC	20 µg vs. 100 µg	4 vs. 4	0.0259 [0.0080, 0.0653] vs. 0.0457 [0.0110, 0.0817]	0.6857	0.9048
Wk3 GC	20 µg vs. 100 µg	2 vs. 3	2.3429 [1.6281, 3.0577] vs. 2.7265 [0.9546, 5.4440]	—	—
Wk9 PB	20 µg vs. 100 µg	2 vs. 3	0.6629 [0.4731, 0.8527] vs. 0.4593 [0.1946, 2.0745]	—	—
**04/*05 or *04/*06**					
Wk4 MBC	20 µg vs. 100 µg	6 vs. 3	0.0066 [0.0031, 0.0140] vs. 0.0062 [0.0055, 0.0092]	0.9048	0.9048
Wk8 MBC	20 µg vs. 100 µg	6 vs. 2	0.0024 [0.0007, 0.0216] vs. 0.0033 [0.0023, 0.0043]	—	—

Supplementary Table 6: Quasi-likelihood version of Akaike's second-order information criterion (QAICc) results. At each sample time point, the four models (Null, Dose, Allele, and Full) are ranked best-to-worst (lower QAICc is better). Each row gives the number of samples analyzed (n), the number of model parameters (K), the log-likelihood (LL) from a Poisson model fit, and the dispersion (Dispersion) from a quasi-Poisson model fit. The QAICc is computed based on these values (see Methods).

Model	n	K	LL	Dispersion	QAICc
**Wk4 MBC**					
Allele	35	3	-954.5112	67.96823	33.63438
Full	35	4	-953.5981	71.07256	36.16783
Dose	35	3	-1241.2651	102.81975	41.70371
Null	35	2	-1353.1930	110.50062	42.45420
**Wk8 MBC**					
Allele	34	3	-467.4507	27.70813	37.91914
Full	34	4	-461.2600	30.04265	40.08632
Dose	34	3	-615.3809	47.22338	47.76716
Null	34	2	-684.2942	44.60637	49.94195
**Wk10 MBC**					
Allele	33	3	-3913.2854	275.82562	34.31452
Full	33	4	-3913.0473	284.73788	36.91383
Dose	33	3	-4507.3975	325.05221	38.48756
Null	33	2	-4997.3051	397.41236	39.50109
**Wk16 MBC**					
Allele	31	3	-1192.3772	95.31918	31.38919
Full	31	4	-1189.6335	97.33573	33.98238
Dose	31	3	-1638.2275	127.00529	40.55027
Null	31	2	-2049.7041	178.03677	46.54474
**Wk3 GC**					
Allele	26	3	-7480.1546	486.41216	37.62126
Full	26	4	-7182.3606	490.01431	39.21966
Null	26	2	-11266.3607	803.67639	50.50554
Dose	26	3	-10976.7429	779.39438	51.89263
**Wk11 GC**					
Null	26	2	-8550.4050	1001.94407	26.62847
Allele	26	3	-7690.3863	705.77005	26.97410
Dose	26	3	-8333.1437	881.98514	28.63592
Full	26	4	-7474.9931	773.55665	29.23106
**Wk9 PB**					
Allele	26	3	-628.4218	45.03017	34.45853
Dose	26	3	-655.0902	46.71709	35.61993
Full	26	4	-613.5097	45.92447	36.62296
Null	26	2	-759.2088	49.71729	37.58511

Supplementary Table 7: Parameter estimates and 95% confidence intervals (CIs) from each model (Allele, Full, Dose, and Null) fit using a quasi-Poisson distribution are organized by sample time point. Abreviated parameter names, Delta, Low and Vacc., are used for the full parameter names defined in the Methods section, Dose delta (100μg - 20μg), Dose=20μg, and Vaccine pooled (a single estimate from the Null model for the vaccine dose groups pooled), respectively.

Parameter	Allele	Full	Dose	Null
**Wk4 MBC**				
*02	0.021 (0.011, 0.031)	0.021 (0.008, 0.033)		
*04	0.006 (0.001, 0.012)	0.006 (0.000, 0.012)		
Low			0.013 (0.001, 0.025)	
Delta		0.001 (-0.012, 0.015)	0.015 (-0.004, 0.034)	
Vacc.				0.022 (0.011, 0.032)
**Wk8 MBC**				
*02	0.011 (0.006, 0.015)	0.010 (0.004, 0.015)		
*04	0.003 (0.001, 0.005)	0.003 (0.000, 0.005)		
Low			0.006 (0.001, 0.012)	
Delta		0.002 (-0.004, 0.008)	0.008 (-0.001, 0.016)	
Vacc.				0.011 (0.007, 0.015)
**Wk10 MBC**				
*02	0.085 (0.056, 0.115)	0.085 (0.041, 0.128)		
*04	0.052 (0.024, 0.079)	0.052 (0.023, 0.080)		
Low			0.086 (0.045, 0.127)	
Delta		0.002 (-0.069, 0.073)	0.060 (-0.008, 0.128)	
Vacc.				0.116 (0.078, 0.153)
**Wk16 MBC**				
*02	0.050 (0.032, 0.069)	0.052 (0.029, 0.076)		
*04	0.012 (0.002, 0.021)	0.012 (0.001, 0.023)		
Low			0.026 (0.005, 0.046)	
Delta		-0.004 (-0.030, 0.023)	0.049 (0.012, 0.087)	
Vacc.				0.053 (0.030, 0.076)
**Wk3 GC**				
*02	1.435 (0.874, 1.996)	1.665 (1.070, 2.261)		
*04	0.506 (0.154, 0.858)	0.584 (0.185, 0.983)		
Low			1.353 (0.500, 2.206)	
Delta		-0.523 (-1.067, 0.020)	0.584 (-0.747, 1.914)	
Vacc.				1.645 (0.970, 2.320)
**Wk11 GC**				
*02	0.602 (0.157, 1.047)	0.630 (0.113, 1.147)		
*04	0.103 (-0.127, 0.333)	0.008 (-0.071, 0.087)		
Low			0.764 (0.116, 1.411)	
Delta		0.147 (-0.631, 0.925)	-0.317 (-1.183, 0.550)	
Vacc.				0.629 (0.154, 1.104)
**Wk9 PB**				
*02	0.386 (0.274, 0.497)	0.268 (-0.009, 0.544)		
*04	0.159 (-0.008, 0.326)	0.115 (-0.064, 0.295)		
Low			0.205 (-0.080, 0.489)	
Delta		0.225 (-0.274, 0.723)	0.508 (0.160, 0.856)	
Vacc.				0.648 (0.463, 0.834)

Supplementary Table 8: Model estimates and 95% confidence intervals (CIs) from the Allele model for each genotype and time point. The estimated percent (and CI) of IgG B cells that are VRC01-class for each genotype are organized by time point. Genotypes containing the *05 and *06 alleles are grouped (e.g., *02/*05 or *02/*06) since the estimated mean response from the Allele model depends only on the count of *02 and *04 alleles. Week 11 germinal center (GC) results are not shown since the Null model ranked better than the Allele model for that time point.

Genotype	Percent (95% CI)
**Wk4 MBC**	
*02/*02	0.042 (0.022, 0.062)
*02/*04	0.028 (0.017, 0.038)
*02/*05 or *02/*06	0.021 (0.011, 0.031)
*04/*04	0.013 (0.002, 0.023)
*04/*05 or *04/*06	0.006 (0.001, 0.012)
**Wk8 MBC**	
*02/*02	0.021 (0.013, 0.029)
*02/*04	0.014 (0.009, 0.018)
*02/*05 or *02/*06	0.011 (0.006, 0.015)
*04/*04	0.006 (0.002, 0.011)
*04/*05 or *04/*06	0.003 (0.001, 0.005)
**Wk10 MBC**	
*02/*02	0.171 (0.112, 0.229)
*02/*04	0.137 (0.100, 0.174)
*02/*05 or *02/*06	0.085 (0.056, 0.115)
*04/*04	0.103 (0.048, 0.158)
*04/*05 or *04/*06	0.052 (0.024, 0.079)
**Wk16 MBC**	
*02/*02	0.101 (0.063, 0.138)
*02/*04	0.062 (0.042, 0.082)
*02/*05 or *02/*06	0.050 (0.032, 0.069)
*04/*04	0.023 (0.004, 0.043)
*04/*05 or *04/*06	0.012 (0.002, 0.021)
**Wk3 GC**	
*02/*02	2.870 (1.747, 3.993)
*02/*04	1.941 (1.321, 2.561)
*02/*05 or *02/*06	1.435 (0.874, 1.996)
*04/*04	1.012 (0.308, 1.716)
*04/*05 or *04/*06	0.506 (0.154, 0.858)
**Wk9 PB**	
*02/*02	0.771 (0.548, 0.994)
*02/*04	0.545 (0.352, 0.738)
*02/*05 or *02/*06	0.386 (0.274, 0.497)
*04/*04	0.318 (-0.016, 0.652)
*04/*05 or *04/*06	0.159 (-0.008, 0.326)

Supplementary Table 9: Ratio and 95% confidence interval (CI) estimates from the Allele model for the relative contribution of alleles *02 and *04 to the post-vaccination VRC01-class B cell frequencies. Week 11 germinal center (GC) results are not shown since the Null model ranked better than the Allele model for that time point.

Visit	Ratio (95% CI)
Wk3 GC	2.836 (0.443, 5.229)
Wk4 MBC	3.278 (0.000, 6.613)
Wk8 MBC	3.259 (0.584, 5.934)
Wk9 PB	2.425 (0.000, 5.124)
Wk10 MBC	1.650 (0.544, 2.757)
Wk16 MBC	4.360 (0.163, 8.557)

Supplementary Table 10: Difference and 95% confidence interval (CI) estimates from the Allele model for the relative contribution of alleles *02 and *04 to the post-vaccination VRC01-class B cell frequencies. Week 11 germinal center (GC) results are not shown since the Null model ranked better than the Allele model for that time point.

Visit	Difference (95% CI)	P Value
Wk3 GC	0.929 (0.226, 1.632)	0.0095
Wk4 MBC	0.015 (0.003, 0.027)	0.0169
Wk8 MBC	0.007 (0.002, 0.012)	0.0030
Wk9 PB	0.227 (0.018, 0.435)	0.0333
Wk10 MBC	0.034 (-0.009, 0.076)	0.1239
Wk16 MBC	0.039 (0.017, 0.061)	0.0006

Supplementary Table 11: Ratio estimates and 95% confidence intervals (CIs) of the relative contributions of alleles *02 and *04 to the naive repertoire. Estimates, 95% CIs and P-values are based on the frequency of mRNA expression and unique HCRD3s among vaccine recipients with homozygous genotypes (*02/*02 and *04/*04) or heterozygous genotype *02/*04 as described in the Methods section.

Frequency	Group	Ratio	95% CI	P-value
mRNA	Homozygote	3.9	3.0 to 5.3	<0.0001
mRNA	Heterozygote	4.2	3.3 to 5.1	0.0001
Unique HCDR3	Homozygote	3.8	3.1 to 4.9	<0.0001
Unique HCDR3	Heterozygote	3.7	3.0 to 4.5	<0.0001

Supplementary Table 12: Ratio of inferred-germline B cell receptor (BCR) assignment among VRC01-class B cells for eight heterozygous IGHV1-2 *02/*04 vaccine recipients combined across time points. The median of these eight ratios is also reported. Four of 873 assignments were ambiguous and were given one half weight to each allele.

dose	ID	*02	*04	Ratio
20µg	046	40.5	5.5	7.364
20µg	077	38.0	13.0	2.923
20µg	100	140.0	18.0	7.778
20µg	187	31.0	15.0	2.067
100µg	062	88.0	21.0	4.190
100µg	064	138.0	19.0	7.263
100µg	112	68.0	4.0	17.000
100µg	193	221.5	12.5	17.720
	Median			7.313

Supplementary Table 13: Ratio of inferred-germline B cell receptor (BCR) assignment among VRC01-class B cells for eight heterozygous IGHV1-2 *02/*04 vaccine recipients organized by time point. The median ratio is also reported for each time point. Four of 873 assignments were ambiguous and were given one half weight to each allele.

dose	ID	*02	*04	Ratio
**Wk4 MBC**				
20µg	046	6.0	1.0	6.000
20µg	077	5.5	1.5	3.667
20µg	100	30.0	4.0	7.500
20µg	187	2.0	5.0	0.400
100µg	062	1.0	2.0	0.500
100µg	064	27.0	5.0	5.400
100µg	112	8.0	2.0	4.000
100µg	193	37.0	3.0	12.333
	Median			4.700
**Wk8 MBC**				
20µg	046	1.0	0.0	Inf
20µg	077	7.5	0.5	15.000
20µg	100	24.0	2.0	12.000
20µg	187	2.0	2.0	1.000
100µg	062	3.0	0.0	Inf
100µg	064	22.0	1.0	22.000
100µg	112	17.0	1.0	17.000
100µg	193	15.0	2.0	7.500
	Median			16.000
**Wk10 MBC**				
20µg	046	27.0	4.0	6.750
20µg	077	1.0	2.0	0.500
20µg	100	16.0	7.0	2.286
20µg	187	18.0	3.0	6.000
100µg	062	11.0	7.0	1.571
100µg	064	18.0	4.0	4.500
100µg	112	11.0	0.0	Inf
100µg	193	34.5	2.5	13.800
	Median			5.250
**Wk16 MBC**				
20µg	046	6.5	0.5	13.000
20µg	077	4.0	1.0	4.000
20µg	100	28.0	5.0	5.600
20µg	187	9.0	5.0	1.800
100µg	062	8.0	5.0	1.600
100µg	064	34.0	2.0	17.000
100µg	112	8.0	0.0	Inf
100µg	193	23.0	3.0	7.667
	Median			6.633

Supplementary Table 13: Ratio of inferred-germline B cell receptor (BCR) assignment among VRC01-class B cells for eight heterozygous IGHV1-2 *02/*04 vaccine recipients organized by time point. The median ratio is also reported for each time point. Four of 873 assignments were ambiguous and were given one half weight to each allele. *(continued)*

dose	ID	*02	*04	Ratio
**Wk3 GC**				
20µg	046			
20µg	077	19.0	8.0	2.375
20µg	100	32.0	0.0	Inf
20µg	187			
100µg	062	49.0	7.0	7.000
100µg	064			
100µg	112	2.0	0.0	Inf
100µg	193	31.0	0.0	Inf
	Median			Inf
**Wk11 GC**				
20µg	046			
20µg	077			
20µg	100	8.0	0.0	Inf
20µg	187			
100µg	062	16.0	0.0	Inf
100µg	064	1.0	0.0	Inf
100µg	112	22.0	0.0	Inf
100µg	193	51.0	0.0	Inf
	Median			Inf
**Wk9 PB**				
20µg	046			
20µg	077	1.0	0.0	Inf
20µg	100	2.0	0.0	Inf
20µg	187			
100µg	062			
100µg	064	36.0	7.0	5.143
100µg	112	0.0	1.0	0.000
100µg	193	30.0	2.0	15.000
	Median			15.000

Supplementary References

52 Scheid, J. F. *et al.* Sequence and structural convergence of broad and potent HIV antibodies that mimic CD4 binding. *Science* **333**, 1633-1637, doi:10.1126/science.1207227 (2011).

53 Huang, J. *et al.* Identification of a CD4-Binding-Site Antibody to HIV that Evolved Near-Pan Neutralization Breadth. *Immunity* **45**, 1108-1121, doi:10.1016/j.immuni.2016.10.027 (2016).

54 Sajadi, M. M. *et al.* Identification of Near-Pan-neutralizing Antibodies against HIV-1 by Deconvolution of Plasma Humoral Responses. *Cell* **173**, 1783-1795 e1714, doi:10.1016/j.cell.2018.03.061 (2018).

55 Jardine, J. G. *et al.* Minimally Mutated HIV-1 Broadly Neutralizing Antibodies to Guide Reductionist Vaccine Design. *PLoS pathogens* **12**, e1005815, doi:10.1371/journal.ppat.1005815 (2016).
