# Supplementary material for: Human immunoglobulin gene allelic variation impacts germline-targeting vaccine priming
Source: NPJ Vaccines. 2024 Mar 11;9:58. doi: 10.1038/s41541-024-00811-5 (PMC11384754; doi:10.1038/s41541-024-00811-5)
Supplement: Supplementary file 6 — Reporting Summary [file 41541_2024_811_MOESM6_ESM.pdf]

nature portfolio

Corresponding author(s): Allan DeCamp
Gunilla Karlsson Hedestam
William R. Schief

Last updated by author(s): 11/22/2023

Reporting Summary

Nature Portfolio wishes to improve the reproducibility of the work that we publish. This form provides structure for consistency and transparency in reporting. For further information on Nature Portfolio policies, see our Editorial Policies and the Editorial Policy Checklist.

Statistics

For all statistical analyses, confirm that the following items are present in the figure legend, table legend, main text, or Methods section.

n/a	Confirmed	
☐	☒	The exact sample size (*n*) for each experimental group/condition, given as a discrete number and unit of measurement
☐	☒	A statement on whether measurements were taken from distinct samples or whether the same sample was measured repeatedly
☐	☒	The statistical test(s) used AND whether they are one- or two-sided<br>*Only common tests should be described solely by name; describe more complex techniques in the Methods section.*
☐	☒	A description of all covariates tested
☐	☒	A description of any assumptions or corrections, such as tests of normality and adjustment for multiple comparisons
☐	☒	A full description of the statistical parameters including central tendency (e.g. means) or other basic estimates (e.g. regression coefficient) AND variation (e.g. standard deviation) or associated estimates of uncertainty (e.g. confidence intervals)
☐	☒	For null hypothesis testing, the test statistic (e.g. F, t, r) with confidence intervals, effect sizes, degrees of freedom and P value noted<br>*Give P values as exact values whenever suitable.*
☒	☐	For Bayesian analysis, information on the choice of priors and Markov chain Monte Carlo settings
☐	☒	For hierarchical and complex designs, identification of the appropriate level for tests and full reporting of outcomes
☐	☒	Estimates of effect sizes (e.g. Cohen's *d*, Pearson's *r*), indicating how they were calculated

Our web collection on statistics for biologists contains articles on many of the points above.

Software and code

Policy information about availability of computer code

Data collection	All data were generated under the phase 1 study IAVI G001 with ClinicalTrials.gov registry number NCT03547245.
Data analysis	See code availability statement in the manuscript.

For manuscripts utilizing custom algorithms or software that are central to the research but not yet described in published literature, software must be made available to editors and reviewers. We strongly encourage code deposition in a community repository (e.g. GitHub). See the Nature Portfolio guidelines for submitting code & software for further information.

Data

Policy information about availability of data

All manuscripts must include a data availability statement. This statement should provide the following information, where applicable:

- Accession codes, unique identifiers, or web links for publicly available datasets
- A description of any restrictions on data availability
- For clinical datasets or third party data, please ensure that the statement adheres to our policy

See data availability statement in the manuscript.

Research involving human participants, their data, or biological material

Policy information about studies with human participants or human data. See also policy information about sex, gender (identity/presentation), and sexual orientation and race, ethnicity and racism.

Reporting on sex and gender	Approximately equal numbers of of participants in IAVI G001 were assigned male and female for sex at birth as reported in Leggat et al., Science, 2022. However, we did not consider sex at birth in our study.
Reporting on race, ethnicity, or other socially relevant groupings	We don not report on race, ethnicity, or other socially relevant groupings in this manuscript.
Population characteristics	We report the IGHV1-2 genotypes and vaccine treatment, high-dose, low-dose, or placebo, of the IAVI G001 participants.
Recruitment	Forty-eight participants were recruited for the IAVI G001 clinical trial with twenty-four participants enrolled at each of two sites: George Washington University (GWU) and Fred Hutchinson Cancer Center (FHCC). As discussed in the manuscript participants were enrolled sequentially into the low- and high-dose groups which resulted in an imbalance in IGHV1-2 genotypes and likely explains the observed dose effect.
Ethics oversight	The trial was conducted under an Investigational New Drug (IND) application submitted to the US Food and Drug Administration, and was carried out in compliance with the protocol filed within the IND. The trial adhered to IAVI standard operating procedures in accordance with the guidelines formulated by the International Committee on Harmonization for Good Clinical Practice in clinical studies, and complied with applicable local standards and regulatory requirements including review and approval by the institutional review boards at FHCC and GWU. Genotyping of the G001 participants was performed under an ethics approval from the National Ethical Review Agency of Sweden (decision no. 2021-01850).

Note that full information on the approval of the study protocol must also be provided in the manuscript.

Field-specific reporting

Please select the one below that is the best fit for your research. If you are not sure, read the appropriate sections before making your selection.

☒ Life sciences ☐ Behavioural & social sciences ☐ Ecological, evolutionary & environmental sciences

For a reference copy of the document with all sections, see nature.com/documents/nr-reporting-summary-flat.pdf

Life sciences study design

All studies must disclose on these points even when the disclosure is negative.

Sample size	No sample size calculation was performed. All available (n=48) participants from the IAVI G001 trial were genotyped while most analyses rely on participants that received active vaccination (n=36). While the original study was not powered specifically for a genotype analysis, all results employed statistical methods that account for the uncertainty due to the available sample size.
Data exclusions	One IGHV1-2 *05/*06 heterozygous participant was excluded from the statistical modeling analysis which relies on *02 and *04 allelic content of each participants IGHV1-2 genotype.
Replication	The personalized genotyping data was carried out with two independent IgM libraries (generated with two different sets of multiplex primers), which both resulted in the same IGHV1-2 genotype.
Randomization	Although participants were randomized to received placebo or vaccine by dose group they were sequentially enrolled into the low- and high-dose arms of the study. This is typical of a first in human dose-escalation study. A key finding of our analysis is that an imbalance in the IGHV1-2 allele content between dose groups better explains the dose effect observed in the trial than dose alone. Controlling for relevant genotype factors in the study of germline-targeting vaccines is an important finding of our work.
Blinding	Study site staff and volunteers were blinded in terms of vaccine versus placebo.

Reporting for specific materials, systems and methods

We require information from authors about some types of materials, experimental systems and methods used in many studies. Here, indicate whether each material, system or method listed is relevant to your study. If you are not sure if a list item applies to your research, read the appropriate section before selecting a response.

Materials & experimental systems

n/a	Involved in the study	
☒	☐	Antibodies
☒	☐	Eukaryotic cell lines
☒	☐	Palaeontology and archaeology
☒	☐	Animals and other organisms
☐	☒	Clinical data
☒	☐	Dual use research of concern
☒	☐	Plants

Methods

n/a	Involved in the study	
☒	☐	ChIP-seq
☒	☐	Flow cytometry
☒	☐	MRI-based neuroimaging

Clinical data

Policy information about clinical studies

All manuscripts should comply with the ICMJE guidelines for publication of clinical research and a completed CONSORT checklist must be included with all submissions.

Clinical trial registration	ClinicalTrials.gov registry number NCT03547245
Study protocol	See Leggat et al., Science, 2022
Data collection	See Leggat et al., Science, 2022. Recruitment and data collection were conducted between the study start and completion dates: 2018-06-15 and 2021-08-13.
Outcomes	Outcomes were defined as exploratory in the protocol
